# Common signatures of differential microRNA expression in Parkinson’s and Alzheimer’s disease brains

**DOI:** 10.1093/braincomms/fcac274

**Published:** 2022-10-28

**Authors:** Valerija Dobricic, Marcel Schilling, Ildiko Farkas, Djordje O Gveric, Olena Ohlei, Jessica Schulz, Lefkos Middleton, Steve M Gentleman, Laura Parkkinen, Lars Bertram, Christina M Lill

**Affiliations:** Lübeck Interdisciplinary Platform for Genome Analytics (LIGA), University of Lübeck, 23562 Lübeck, Germany; Lübeck Interdisciplinary Platform for Genome Analytics (LIGA), University of Lübeck, 23562 Lübeck, Germany; Multiple Sclerosis and Parkinson’s Tissue Bank, Imperial College London, London W12 0NN, UK; Multiple Sclerosis and Parkinson’s Tissue Bank, Imperial College London, London W12 0NN, UK; Lübeck Interdisciplinary Platform for Genome Analytics (LIGA), University of Lübeck, 23562 Lübeck, Germany; Lübeck Interdisciplinary Platform for Genome Analytics (LIGA), University of Lübeck, 23562 Lübeck, Germany; Ageing and Epidemiology Unit (AGE), School of Public Health, Imperial College London, London W6 8RF, UK; Public Health Directorate, Imperial College NHS Healthcare Trust, London W6 8RF, UK; Department of Brain Sciences, Hammersmith Hospital campus, Imperial College London, London W12 0HS, UK; Nuffield Department of Clinical Neurosciences, Oxford Parkinson’s Disease Centre, University of Oxford, Oxford OX3 9DU, UK; Lübeck Interdisciplinary Platform for Genome Analytics (LIGA), University of Lübeck, 23562 Lübeck, Germany; Department of Psychology, University of Oslo, 0373 Oslo, Norway; Lübeck Interdisciplinary Platform for Genome Analytics (LIGA), University of Lübeck, 23562 Lübeck, Germany; Ageing and Epidemiology Unit (AGE), School of Public Health, Imperial College London, London W6 8RF, UK; Institute of Epidemiology and Social Medicine, University of Münster, 48149 Münster, Germany

**Keywords:** Parkinson’s disease, Alzheimer’s disease, miRNA, expression, brain

## Abstract

Dysregulation of microRNA gene expression has been implicated in many neurodegenerative diseases, including Parkinson’s disease. However, the individual dysregulated microRNAs remain largely unknown. Previous meta-analyses have highlighted several microRNAs being differentially expressed in post-mortem Parkinson’s disease and Alzheimer's disease brains versus controls, but they were based on small sample sizes. In this study, we quantified the expression of the most compelling Parkinson’s and Alzheimer’s disease microRNAs from these meta-analyses (‘candidate miRNAs’) in one of the largest Parkinson’s/Alzheimer’s disease case–control post-mortem brain collections available (*n* = 451), thereby quadruplicating previously investigated sample sizes. Parkinson’s disease candidate microRNA hsa-miR-132-3p was differentially expressed in our Parkinson’s (*P* = 4.89E−06) and Alzheimer’s disease samples (*P* = 3.20E−24) compared with controls. Alzheimer’s disease candidate microRNAs hsa-miR-132-5p (*P* = 4.52E−06) and hsa-miR-129-5p (*P* = 0.0379) were differentially expressed in our Parkinson’s disease samples. Combining these novel data with previously published data substantially improved the statistical support (α = 3.85E−03) of the corresponding meta-analyses, clearly implicating these microRNAs in both Parkinson’s and Alzheimer’s disease. Furthermore, hsa-miR-132-3p/-5p (but not hsa-miR-129-5p) showed association with α-synuclein neuropathological Braak staging (*P* = 3.51E−03/*P* = 0.0117), suggesting that hsa-miR-132-3p/-5p play a role in α-synuclein aggregation beyond the early disease phase. Our study represents the largest independent assessment of recently highlighted candidate microRNAs in Parkinson’s and Alzheimer’s disease brains, to date. Our results implicate hsa-miR-132-3p/-5p and hsa-miR-129-5p to be differentially expressed in both Parkinson’s and Alzheimer’s disease, pinpointing shared pathogenic mechanisms across these neurodegenerative diseases. Intriguingly, based on publicly available high-throughput sequencing of RNA isolated by cross-linking immunoprecipitation data, hsa-miR-132 may interact with *SNCA* messenger RNA in the human brain, possibly pinpointing novel therapeutic approaches in fighting Parkinson’s disease.

## Teaser

Dobricic *et al*. quantified the expression of 10 candidate microRNAs for Parkinson’s disease and Alzheimer’s disease in one of the largest Parkinson’s disease/Alzheimer’s disease case–control brain collections available (*n* = 451). They observed differential expression of hsa-miR-132-3p, hsa-miR-132-5p and hsa-miR-129-5p in both Parkinson’s disease and Alzheimer’s disease, pinpointing possible shared pathogenic mechanisms across these diseases.

## Graphical Abstract

**Graphical Abstract:**
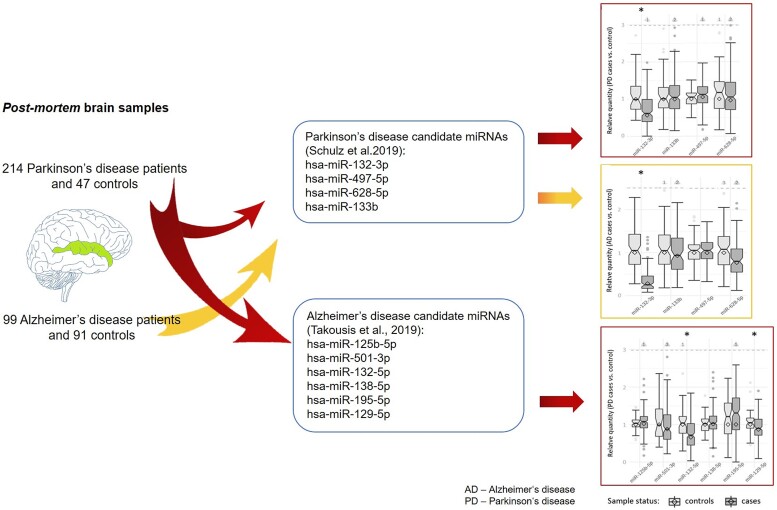

## Introduction

Parkinson’s disease is—after Alzheimer’s disease—the second most common neurodegenerative disorder affecting 1–2% of the general population over the age of 60 years with increasing incidences in industrialized populations.^1,2^ Currently, there is no curative or preventive therapy available for Parkinson’s disease, which is in part attributable to our lack of understanding its aetiology. More than 90% of the disease is genetically complex, i.e. it is determined by a combination and likely interaction of multiple genetic, environmental, lifestyle and other intrinsic risk factors.^3^ While genome-wide association studies (GWASs) have identified ∼90 independent genetic risk variants in Parkinson’s disease^4^ and several environmental and lifestyle variables have been described as being associated with Parkinson’s disease,^5^ a substantial fraction of the disease variance remains unexplained. In this context—as for other neurodegenerative diseases—epigenetic mechanisms have been suggested to play a significant role in the molecular architecture of Parkinson’s disease. One proposed mechanism is the action of microRNAs (miRNAs) (reviewed in e.g. ref.^3,6^). MiRNAs are small non-coding RNAs that bind to messenger RNAs (mRNAs) and thereby inhibit their translation into proteins.^7^ However, despite an increasing amount of data published on the possible role of miRNAs in Parkinson’s disease pathogenesis (reviewed in ref.^8,9^), the interpretation of the individual findings has often been impeded by inconclusive or even discrepant results across studies. This can at least partly be attributed to the use of small sample sizes,^10^ which is of particular relevance in expression studies of post-mortem brain samples due to the paucity of available biomaterial. In order to identify miRNAs of potential relevance in Parkinson’s disease, our group recently performed systematic meta-analyses on all published miRNA gene expression studies comparing post-mortem brains of Parkinson’s disease patients with control individuals; four miRNAs (i.e. hsa-miR-132-3p, -497-5p, -133b, -628-5p) showed significant evidence for differential miRNA expression changes in Parkinson’s disease versus controls.^10^ Despite having merged all available data in the field, the sample sizes for the meta-analyses still remained comparatively small [median *n* = 88 derived from a median of three data sets, interquartile range (IQR): 87–98]. Similar observations were made in Alzheimer’s disease, where we performed similar meta-analyses across all published differential miRNA expression studies^11^ with a median *n* = 42.5 derived from a median of three data sets (IQR: 23–85).^11^ We recently performed an independent validation study in Alzheimer’s disease brain tissue, in which we confirmed differential expression for four out of six candidate miRNAs (miR-129-5p, miR-132-5p, miR-138-5p and miR-195-5p).^12^

**Figure 1:**
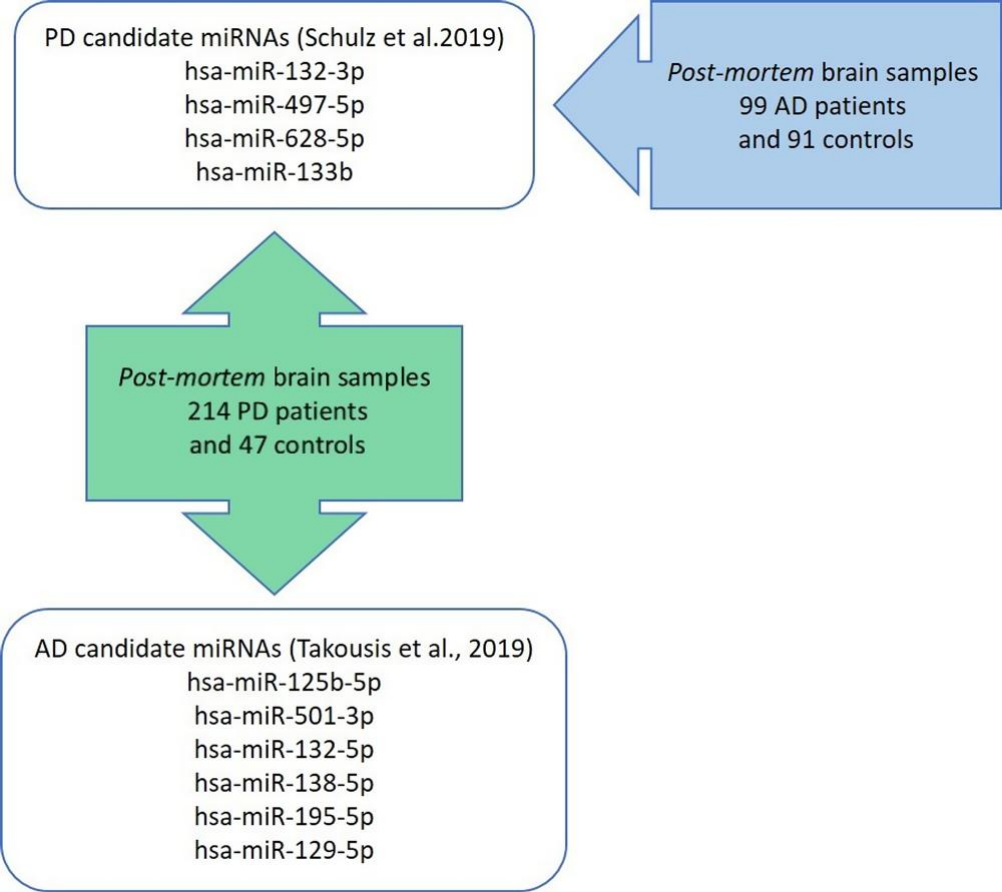
**Overview of the study design.** PD, Parkinson’s disease; AD, Alzheimer’s disease.

In order to independently assess the role of the four candidate miRNAs in Parkinson’s disease mentioned above, we investigated their expression in 261 post-mortem brain samples including 214 Parkinson’s disease patients and 47 controls, thereby effectively quadruplicating previously investigated sample sizes. Furthermore, we assessed common signatures of differential miRNA expression across neurodegenerative diseases by investigating the same four miRNAs in post-mortem brain samples of 190 Alzheimer’s disease patients and controls and by assessing expressional changes of six of the most promising Alzheimer’s disease miRNAs^11^ in Parkinson’s disease brain samples (Fig. 1). In addition, we assessed the association of differentially expressed miRNAs with neuropathological Braak staging in Parkinson’s disease brains.

## Materials and methods

### Brain samples

To investigate differential miRNA gene expression in Parkinson’s disease and Alzheimer’s disease brains, we used superior temporal gyrus (STG) sections for this study because this brain region has been identified as one of the first common regions to be affected in Parkinson’s disease^13^ and Alzheimer’s disease.^14^ All Parkinson’s disease/Alzheimer’s disease/control brain samples available at the time of the study were included. For Parkinson’s disease, snap-frozen STG sections (Brodmann area BA21) of 235 deceased patients with a clinical and neuropathologically confirmed diagnosis of Parkinson’s disease and 47 controls were provided by the Parkinson’s UK Brain Bank at Imperial College London. Staging of α-synuclein and tau pathology was conducted according to the BrainNet Europe Consortium criteria.^15,16^ The presence and distribution of α-synuclein and tau deposits were assessed using immunohistochemistry, with antibodies specific for a-synuclein (mouse anti-α-synuclein, BD Transduction Laboratories, Franklin Lakes, NJ, USA) and hyperphosphorylated tau (AT8, Invitrogen, Waltham, MA, USA). For Alzheimer’s disease, we used the same snap-frozen STG sections (Brodmann area BA21) of 99 Alzheimer’s disease patients and 91 elderly control individuals provided by the Oxford Brain Bank that were analysed in our previous validation study (see ref.^12^). In brief, Alzheimer’s disease patients and controls were part of the longitudinal, prospective Oxford Project to Investigate Memory and Aging (OPTIMA) and underwent a standard battery of clinical and neuropsychological tests.^17^ The pathological diagnosis of Alzheimer’s disease was made using the CERAD/NIH criteria and Braak Alzheimer’s disease staging.^18–20^ All participants had given prior written informed consent for the brain donation. The tissue bank activities of the Parkinson’s UK Brain Bank were approved by the Ethics Committee for Wales (ref 18/WA/0238), and the Oxford Brain Bank activities were approved by the Ethics Committee of the University of Oxford (ref 15/SC/0639).

### miRNA extraction and qPCR analysis

Total RNA (containing miRNAs and other remaining RNAs) was extracted from brain tissue in one sample using the mirVana isolation kit (Thermo Fisher Scientific, Waltham, MA, USA) following the manufacturer’s instructions without changes to the protocol. This allowed us to quantify RNA integrity numbers (RINs) (see below) in the total RNA samples used for miRNA quantification (measuring RIN in miRNA-enriched RNA fractions is not possible) and to utilize TaqMan Advanced miRNA Assays, which are optimized for the input of total RNA. For each extraction, we used 25 mg of snap-frozen tissue. In order to minimize potential batch effects, patients and controls were randomly mixed before each extraction procedure. Residual DNA in the RNA samples was removed using DNase (TURBO DNA-free kit, Thermo Fisher Scientific). RNA quantity was assessed using NanoDrop 2000 (Thermo Fisher Scientific), and RNA quality was assessed by determining RIN using the Agilent 2100 Bioanalyzer system with the Agilent RNA 6000 Nano Chip Kit (Agilent Technologies, Santa Clara, CA, USA).

Reverse transcription reactions were performed on 10 ng of total RNA using the TaqMan Advanced miRNA cDNA Synthesis Kit (Thermo Fisher Scientific), as per the manufacturer’s instructions without changes to the protocol. In the Parkinson’s disease case–control brain samples we quantified a total of 10 miRNAs: these included all 4 miRNAs showing significant differential expression in brain tissue of Parkinson’s disease patients and controls in our recent meta-analysis,^10^ i.e. hsa-miR-132-3p (MIMAT0000426), hsa-miR-497-5p (MIMAT0002820), hsa-miR-628-5p (MIMAT0004809) and hsa-miR-133b (MIMAT0000770). In addition, we quantified the six most compelling miRNAs from our recent meta-analyses of differential miRNA expression studies in Alzheimer’s disease,^11^ which were also assessed in Alzheimer’s disease brain tissue in our recent validation study.^12^ Specifically, we selected miRNAs that were among the top 10 most significantly associated miRNAs in Alzheimer’s disease brains in that study^11^ and, in addition, showed little between-study heterogeneity (i.e. ≥80% of the datasets showed the same direction of effect): hsa-miR-125b-5p (MIMAT0000423), hsa-miR-501-3p (MIMAT0004774), hsa-miR-132-5p (MIMAT0004594), hsa-miR-138-5p (MIMAT0000430), hsa-miR-195-5p (MIMAT0000461) and hsa-miR-129-5p (MIMAT0000242). Furthermore, in the Alzheimer’s disease case–control brain samples, we assessed the expression of the four Parkinson’s disease miRNAs from our previous study^10^ (listed above). As endogenous controls, alongside all samples, we used hsa-miR-423-5p (suggested as endogenous control by ThermoFisher, Inc.) for Parkinson’s disease and hsa-miR-423-5p and hsa-let-7b-5p for Alzheimer’s disease. All assays were run in a pre-spotted 384-well format on a QuantStudio-12K-Flex system (Thermo Fisher Scientific). Samples were measured in triplicate. Raw data analysis was performed using the ExpressionSuite Software v1.2 (Thermo Fisher Scientific). Samples for which either the endogenous control assay (*n* = 10) or ≥4 target miRNA assays failed (*n* = 11) were excluded from all subsequent analyses. In addition, for the samples passing sample quality control (QC) (214 Parkinson’s disease patients, 47 controls; 99 Alzheimer’s disease patients, 91 controls) individual assays with differences in *C_t_* value (Δ*C_t_*) > 0.5 across the triplicate measurements were excluded. Samples not passing QC or with otherwise missing data were excluded. The exact number of samples for each assay included in all subsequent statistical analyses is given in Table 1.

**Table 1:** Differential expression analysis of Parkinson’s disease candidate miRNAs in brains of Parkinson’s disease and Alzheimer’s disease patients and controls

Parkinson’s disease candidate miRNA	Parkinson’s disease				Alzheimer’s disease			
	Relative quantity (95% CI)	Effect size (±SE)	*P*-value	*N* (Parkinson’s disease cases, controls)	Relative quantity (95% CI)	Effect size (±SE)	*P*-value	*N* (Alzheimer’s disease cases, controls)
**hsa-miR-132-3p**	0.567 (0.507, 0.635)	−0.708 (0.151)	**4.89E−06**	253 (211, 42)	0.282 (0.248, 0.320)	−1.21 (0.102)	**3.23E−24**	190 (99, 91)
hsa-miR-133b	0.999 (0.935, 1.067)	−0.0282 (0.169)	0.868	248 (204, 44)	0.927 (0.837, 1.026)	0.0879 (0.168)	0.602	186 (96, 90)
hsa-miR-497-5p	1.050 (0.994, 1.110)	0.0556 (0.159)	0.726	251 (206, 45)	0.998 (0.940, 1.060)	0.239 (0.163)	0.143	190 (99, 91)
hsa-miR-628-5p	0.965 (0.882, 1.055)	−0.0902 (0.153)	0.556	246 (203, 43)	0.769 (0.688, 0.858)	−0.226 (0.151)	0.137	189 (98, 91)

This table displays the statistical results of the differential gene expression analyses of Parkinson’s disease candidate miRNAs in post-mortem brain samples of 214 Parkinson’s disease patients and 47 controls and of 99 Alzheimer’s disease patients and 91 controls, respectively. *P*-values displayed in bold highlight nominally significant (*α* = 0.05) differential expression results. SE, standard error; *N*, number.

### Statistical analysis

Comparisons of age, post-mortem intervals (PMIs), RIN values and RNA absorbances between patients and control samples were performed by Welch’s *t*-test, and, in case of non-normality, by Wilcoxon rank-sum test, and the comparison of sex distributions was compared by the *χ*^2^ test using R (https://www.R-project.org/). Differential gene expression analysis was performed by (independently) fitting a (Gaussian) generalized linear model (GLM) to predict each candidate miRNA expression (measured by qPCR Δ*C_t_*; scaled and centred) from disease status (case versus control) and potential confounding factors [RIN, age at death, PMI (all scaled and centred) and sex]. Significance was assessed by testing against the null hypothesis that the disease status does not contribute (i.e. zero-weight) to the model (two-sided *z*-test). The Type 1 error rate was set to α = 0.05 for this validation approach (see below for our conservative multiple testing correction of the corresponding meta-analyses).

Furthermore, we utilized Parkinson’s disease cases to train GLMs predicting gene expression of the top miRNAs hsa-miR-132-3p/-5p and hsa-miR-129-5p based on α-synuclein and tau Braak staging (see above) as continuous (scaled and centred) separate outcomes. Covariate adjustments were identical to the case versus control analyses. While we performed six tests in this arm of the study (testing α-synuclein and tau Braak staging for three miRNAs each), we note that levels for hsa-miR-132-3p and hsa-miR-132-5p are highly correlated (Pearson’s *r* = 0.93, *P* = 2.2E−16). Thus, the Type 1 error rate for this arm of the study was set to α = 0.05/4 = 0.0125 using Bonferroni correction for four independent tests.

### Literature search and meta-analyses

We assessed the overall evidence for differential expression of the 10 Parkinson’s disease and Alzheimer’s disease candidate miRNAs in Parkinson’s disease brains by updating our earlier meta-analyses.^10^ To this end, we included the data on these 10 miRNAs reported in the previous study,^10^ the molecular data generated in our current study and other recently published data. For the literature update, we applied the exact same methodology as described previously^10^ and included all studies published until 1 December 2021. Similarly, the meta-analyses performed in Alzheimer’s disease for the four Parkinson’s disease candidate miRNAs^11^ were updated using the same approach by building on the original meta-analysis data by Takousis *et al*.^11^

To correct for multiple testing we used Bonferroni’s method and adjusted for 13 independent tests: 10 miRNAs were tested for Parkinson’s disease (nine of which were independent, see above), and four uncorrelated miRNAs were tested for Alzheimer’s disease, resulting in a two-sided study-wide α = 0.05/13 = 3.85E−03 for the meta-analysis results. In addition, we pre-defined the requirement that a miRNA was to be considered as associated with Parkinson’s disease and Alzheimer’s disease, respectively, only if it passed the study-wide Type 1 error rate of a two-sided α = 3.85E−03 in the meta-analyses *and* if statistical significance improved in the meta-analysis combining previously published and newly generated data in comparison to the results described in Schulz *et al*.^10^ or Takousis *et al*.,^11^ respectively.

## Results

### Demographics and RNA quality assessments

The effective data set analysed in this study comprised post-mortem STG brain samples of 214 patients and 47 controls for Parkinson’s disease (Supplementary Table S1) and of 99 patients and 91 controls for Alzheimer’s disease (Supplementary Table S2). The age at death, sex distribution and PMI until the collection of brain samples did not show significant differences between Parkinson’s disease patients and controls. For Alzheimer’s disease samples, there was a nominally significant difference in the age-at-death distribution and significant associations of RIN and RNA absorbance values with disease status (Supplementary Table S2). However, a comparison of raw expression data in the Alzheimer’s disease data set showed that the distribution of *C_t_* values was similar for samples with lower (RIN < 5) versus higher RIN values (RIN ≥ 5) suggesting that sample quality has not substantially impacted our results (Supplementary Fig. S1). To adjust for potential residual confounding due to these variables we included them as covariates in the linear regression model for both Parkinson’s disease and Alzheimer’s disease (see below).

### Differential miRNA expression analysis in STG

**Figure 2:**
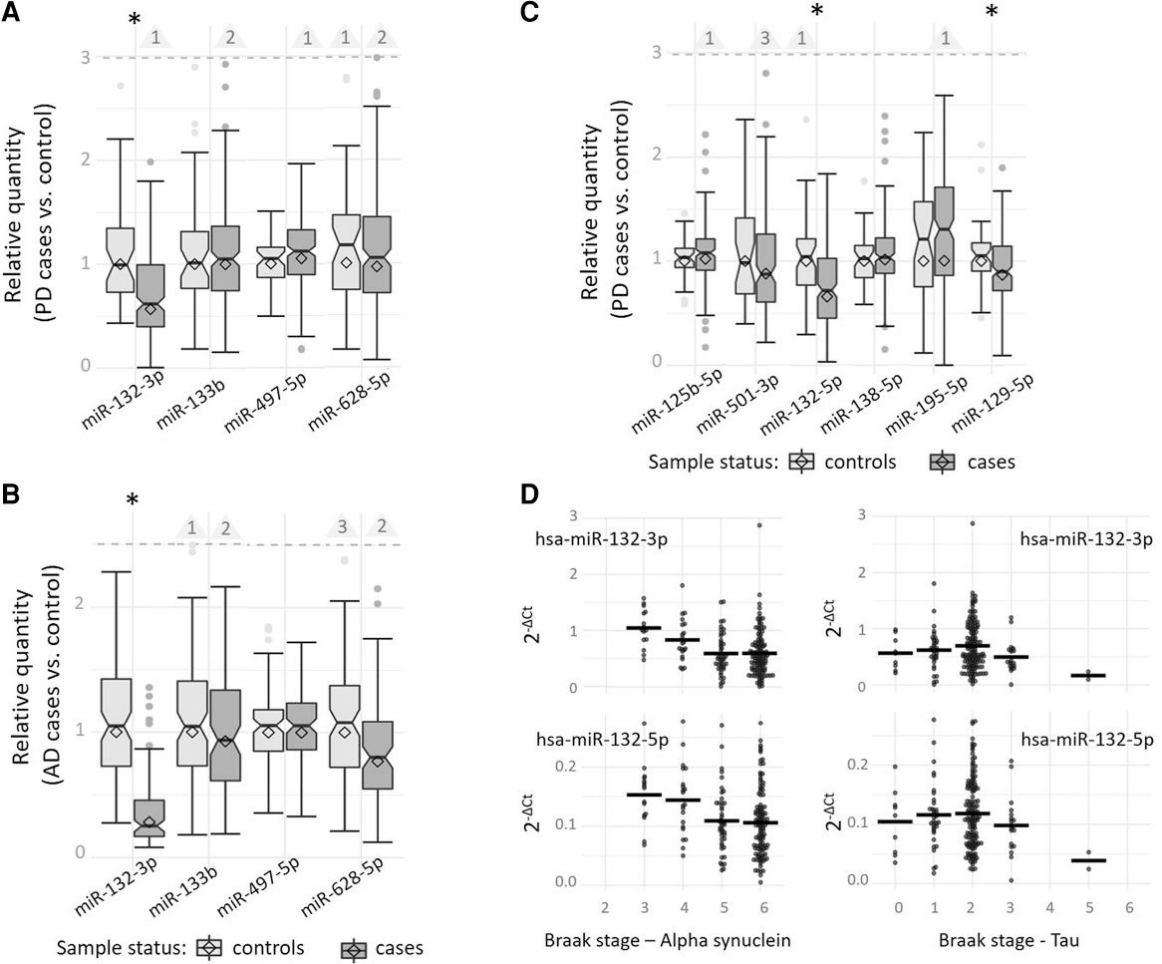
**Expression of candidate miRNAs in brain samples of Parkinson’s disease and Alzheimer’s disease patients versus controls and dependent on the neuropathological α-synuclein and tau Braak staging.** This box and whisker plots display the relative quantity of Parkinson’s disease candidate miRNAs in post-mortem brain samples of the STG of 214 Parkinson’s disease patients versus 47 controls (**A**), the relative quantity of Parkinson’s disease candidate miRNAs in post-mortem brain samples of 99 Alzheimer’s disease patients versus 91 controls (**B**), and the relative quantity of Alzheimer’s disease candidate miRNAs in post-mortem brain samples of the STG of 214 Parkinson’s disease patients versus 47 controls (**C**). Differential gene expression was analysed using a GLM adjusting for RIN, age at death, PMIs and sex. The relative quantity of miRNA expression was calculated using the ΔΔ*C_t_* method; diamonds represent the mean expression (cases relative to controls) based on the ΔΔ*C_t_* method ($\mathrm{relative}\;\mathrm{quantity}=2^{\left(-(dC_{t}\mathrm{cases}\text{–}dC_{t}\mathrm{controls})\right)}$). Horizontal lines represent median values of the corresponding sample-specific values (individual *dC_t_* values normalized to the mean of the control samples), boxes represent IQRs and whiskers extend to the maximum observed value within 1.5 × the IQR; values outside this range but below the dashed line are depicted as dots. Outliers exceeding the dashed line are not shown (for scaling purposes) but counted and indicated by the numbers in the triangles. The box notches indicate the corresponding 95% confidence intervals (CIs). *Nominally statistically significant difference (α = 0.05). (**D**) This plot displays the gene expression of hsa-miR-132-3p and hsa-miR-132-5p normalized to endogenous control assay (*dC_t_*) dependent on α-synuclein and tau Braak staging in post-mortem brain samples of 213 Parkinson’s disease patients utilizing a GLM adjusting for RIN, age at death, PMIs and sex. Horizontal lines indicate the mean of the group.

Quantification of the four Parkinson’s disease candidate miRNAs confirmed that hsa-miR-132-3p was strongly and significantly downregulated in our large independent collection of Parkinson’s disease brain samples compared with controls (*P* = 4.89E−06; Table 1, Fig. 2A). The other three miRNAs (hsa-miR-133b, miRNAs hsa-miR-497-5p and hsa-miR-628-5p) did not show evidence for differential expression in Parkinson’s disease (Table 1, Fig. 2A). Assessment of these four Parkinson’s disease candidate miRNAs in our Alzheimer’s disease dataset revealed that hsa-miR-132-3p was strongly downregulated not only in Parkinson’s disease but also in Alzheimer’s disease (*P* = 3.20E−24; Fig. 2B). The remaining three Parkinson’s disease candidate miRNAs did not show significant differential expression in Alzheimer’s disease samples, either, similar to our observation in Parkinson’s disease samples (Table 1, Fig. 2B).

To further investigate cross-disease miRNA expression changes, we also quantified the six previously described Alzheimer’s disease candidate miRNAs in our Parkinson’s disease data set. These analyses revealed that hsa-miR-132-5p was significantly downregulated in Parkinson’s disease brains compared with controls (*P* = 4.52E−06, Table 2, Fig. 2C). Further analyses showed that this miRNA was highly correlated with hsa-miRNA-132-3p in our data (Pearson’s *r* = 0.93, *P* = 2.2E−16, also see Materials and methods). In addition, the Alzheimer’s disease candidate miRNA miR-129-5p showed evidence for significant downregulation in Parkinson’s disease samples (*P* = 0.0379). None of the other four Alzheimer’s disease candidate miRNAs (hsa-miR-125b-5p, hsa-miR-138-5p, hsa-miR-195-5p and hsa-miR-501-3p) showed statistically significant differential expression in Parkinson’s disease data set.

**Table 2:** Differential expression analysis of Alzheimer’s disease candidate miRNAs in brain samples of Parkinson’s disease patients and controls

Alzheimer’s disease candidate miRNA	Relative quantity (95% CI)	Effect size (±SE)	*P*-value	*N* (Parkinson’s disease cases, controls)
hsa-miR-125b-5p	1.021 (0.975, 1.069)	0.0309 (±0.155)	0.842	244 (200, 44)
hsa-miR-501-3p	0.880 (0.806, 0.960)	−0.186 (±0.183)	0.312	197 (162, 35)
**hsa-miR-132-5p**	0.659 (0.609, 0.714)	−0.705 (±0.15)	**4.52E−06**	255 (209, 46)
hsa-miR-138-5p	1.014 (0.971, 1.059)	0.0474 (±0.161)	0.769	254 (208, 46)
hsa-miR-195-5p	1.003 (0.871, 1.155)	−0.0875 (±0.152)	0.565	244 (198, 46)
**hsa-miR-129-5p**	0.868 (0.821, 0.917)	−0.333 (±0.159)	**0**.**0379**	249 (203, 46)

This table displays the statistical results of the differential gene expression analyses of Alzheimer’s disease candidate miRNAs in post-mortem brain samples of 214 Parkinson’s disease patients and 47 controls. *P*-values displayed in bold highlight nominally significant (α = 0.05) differential expression results. SE, standard error; *N*, number.

To assess whether differential expression of hsa-miR-132-3p/5p and/or hsa-miR-129-5p may be mediated by disease risk variants (acting in *cis* on hsa-miR-132-3p/5p and/or hsa-miR-129-5p expression), we consulted the most recent and largest Parkinson’s disease^4^ and Alzheimer’s disease^21^ GWAS: none of these studies directly reported *MIR129-1*, *MIR129-2*, or *MIR132* as risk genes.^4,21^ However, one of the genome-wide significantly (α = 5 × 10^−8^) associated Parkinson’s disease risk SNPs (rs35048651) is located ∼322 kb upstream from *MIR132* raising the possibility that this SNP may act as a miRNA eQTL. While we did not have genotype data for rs35048651, we were able to analyse a proxy variant in perfect linkage disequilibrium (i.e. rs4790286, *r*^2^ = 1.0 to rs35048651). Using this variant as a predictor in eQTL analyses did not support the hypothesis that this Parkinson’s disease GWAS signal relates to the expression of hsa-miR-132-3p/5p in 85 post-mortem human control brains from Oxford (*P* = 0.490/*P* = 0.360; Schilling *et al.*, in preparation). Thus, we did not find strong evidence for miRNA eQTL effects that could explain the differential miRNA expression of hsa-miR-132-3p/5p and/or hsa-miR-129-5p observed in this study. However, this risk SNP also represents an inframe deletion variant of a trinucleotide Glutamine repeat in *WDR81*, which may represent a better candidate gene.

### Meta-analysis of novel miRNA expression results with published evidence

To assess the overall evidence for differential expression of the 10 Parkinson’s disease and Alzheimer’s disease candidate miRNAs tested in Parkinson’s disease in this study, we added our novel molecular results to the meta-analyses of our previous systematic review,^10^ resulting in a substantial increase of the sample sizes available for the respective miRNAs, by ∼4-fold on average. At the same time, we added the results from two new smaller studies,^22,23^ which were published since our initial data freeze. This increase in available data allowed us to meta-analyse two Alzheimer’s disease candidate miRNAs (hsa-miR-132-5p and hsa-miR-195-5p), for the first time in Parkinson’s disease (Table 3). Previously, these two miRNAs lacked sufficient data for meta-analysis.^10^

**Table 3:** Meta-analysis results for Parkinson’s disease and Alzheimer’s disease candidate miRNAs in Parkinson’s disease brain samples

Parkinson’s disease/Alzheimer’s disease candidate miRNA	Previous Parkinson’s disease meta-analysis (Schulz *et al*.^10^)					Updated Parkinson’s disease meta-analysis including current study				
	Expr. per data set	Overall expr.	*P*-value	*N* (Parkinson’s disease cases, controls)	*N* studies	Expr. per data set	Overall expr.	*P*-value	*N* (Parkinson’s disease cases, controls)	*N* studies
**hsa-miR-132-3p**	−, −, −	Down	6.37E−05	84 (41, 43)	3	−, −, −, −, −, −*	Down	**6.46E**−**09**	378 (275, 103)	6
hsa-miR-497-5p	+, +, +, +	Up	1.35E−04	119 (65, 54)	4	+, +, +, +, −, +*	Up	0.007	386 (279, 107)	6
hsa-miR-133b	−, −, −, −	Down	1.90E−04	90 (45, 45)	4	−, −, −, −, +, −*	Down	0.035	363 (264, 99)	6
hsa-miR-628-5p	−, +, +	Up	1.67E−04	88 (44, 44)	3	−, +, +, +, −*	Up	0.066	350 (255, 95)	5
hsa-miR-125b-5p	−, −, +, +	Up	0.420	98 (48, 50)	4	−, −, +, +, −, −, +*	Down	0.948	383 (271, 112)	7
hsa-miR-501-3p	+, −, +	Down	0.324	80 (38, 42)	3	+, −, +, +, −*	Down	0.181	293 (208, 85)	5
**hsa-miR-132-5p**	n.a.	n.a.	n.a.	n.a.	n.a.	−, −, −, −*	Down	**1.09E**−**09**	342 (252, 90)	4
hsa-miR-138-5p	−, +, −	Up	0.474	88 (44, 44)	3	−, +, −, −, +*	Up	0.540	358 (260, 98)	5
hsa-miR-195-5p	n.a.	n.a.	n.a.	n.a.	n.a.	−, −, −, −*	Down	0.547	334 (243, 91)	4
**hsa-miR-129-5p**	−, −, +	Down	7.74E−04	105 (58, 47)	3	−, −, +, +, −*	Down	**3.30E**−**04**	370 (269, 101)	5

This table displays the meta-analysis results combining published gene expression data with those generated in the current study in post-mortem brain samples of Parkinson’s disease patients and controls for the four Parkinson’s disease (upper part) and six Alzheimer’s disease (lower part) candidate miRNAs assessed in our current study. Meta-analyses were generated by combining all gene expression data published by Schulz *et al.*,^10^ as well as those newly published and those newly generated in this study (for details see Materials and methods). *P*-values displayed in bold highlight study-wide significant meta-analysis results (α = 3.85E−03) that also improved in significance in comparison to the previous meta-analysis. Expr, expression; *N*, number. *Association result from the brain samples newly analysed in this study.

The updated meta-analyses showed that hsa-miR-132-3p is significantly differentially expressed in Parkinson’s disease (Table 3). The statistical support of this association is much stronger now than in our previous study (*P* = 6.46E−09 compared with *P* = 6.37E−05, respectively, Table 3). Second, as expected, the remaining three Parkinson’s disease candidate miRNAs, which were not validated in our novel Parkinson’s disease brain data set, no longer reached study-wide significance (α = 3.85E−03) in the updated meta-analyses (Table 3). Third, meta-analyses of the six Alzheimer’s disease candidate miRNAs revealed novel and study-wide significant differential expression for hsa-mir-132-5p (*P* = 1.09E−09) and for hsa-mir-129-5p in Parkinson’s disease brains (*P* = 3.30E−04, Table 3). Finally, we observed study-wide significant differential expression of hsa-miR-132-3p in Alzheimer's disease brain samples (1.69E−17 compared with *P* = 0.03 in the previous meta-analysis)^11^ but not of the three other Parkinson’s disease candidate miRNAs (hsa-miR-133b, hsa-miR-497-5p and hsa-miR-628-5p; Table 4).

**Table 4:** Meta-analysis results for Parkinson’s disease candidate miRNAs in Alzheimer’s disease brain samples

Parkinson’s disease candidate miRNA	Previous Alzheimer’s disease meta-analysis (Takousis *et al*.^11^)					Updated Alzheimer’s disease meta-analysis including current study				
	Expr. per data set	Overall expr.	*P*-value	*N* (Alzheimer’s disease cases, controls)	*N* studies	Expr. per dataset	Overall expr.	*P*-value	*N* (Alzheimer’s disease cases, controls)	*N* studies
**hsa-miR-132-3p**	−, −, +, +, −, −, −, −, −, −, −, −, −, −, +, +, −, +, +	Down	0.026	792 (481, 311)	19	−, −, +, +, −, −, −, −, −, −, −, −, −, −, +, +, −, +, +, +, −, −, −*	Down	**1.69E−17**	1768 (1044, 724)	23
hsa-miR-497-5p	−, +, −, −,	Down	0.437	37 (19, 18)	4	−, +, −, −, +*	Up	0.0961	227 (118, 109)	5
hsa-miR-133b	n.a.	n.a.	n.a.	n.a.	n.a.	−, +, +, +*	Up	0.0344	269 (131, 138)	4
hsa-miR-628-5p	n.a.	n.a.	n.a.	n.a.	n.a.	−, +, −*	Down	0.162	200 (103, 97)	3

This table displays the meta-analysis results combining published gene expression data and those generated in the current study in post-mortem brain samples of Alzheimer’s disease patients and controls for the four Parkinson’s disease candidate miRNAs. Meta-analyses were generated by combining all gene expression data published Takousis *et al.*,^11^ as well as those newly published and those newly generated in this study (for details, see Materials and methods). *P*-values displayed in bold highlight study-wide significant meta-analysis results (*α* = 3.85E−03) that also improved in significance in comparison to the previous meta-analysis. Expr, expression; *N*, number. *Association result from the brain samples newly analysed in this study.

### Linear regression analyses of hsa-mir-132-3p/-5p and hsa-mir-129-5p on Parkinson’s disease neuropathology

To gauge at which phase in the disease trajectory the two new Parkinson’s disease-linked miRNAs hsa-miR-132-3p/-5p and hsa-miR-129-5p show the strongest degree of differential expression, we assessed their association with α-synuclein and tau Braak staging in the brains of Parkinson’s disease patients (for the distribution of Braak and tau staging in the Parkinson’s disease brains, see Supplementary Table S1). Interestingly, hsa-miR-132-3p/-5p expression showed statistically significant association (α = 0.0125) with α-synuclein staging (*P* = 3.51E−03 for hsa-miR-132-3p, *P* = 0.0117 for hsa-miR-132-5p) but no or only nominally statistically significant association with tau staging (*P* = 0.119 and *P* = 0.0235, respectively; Fig. 2D, Supplementary Table S3). In contrast, hsa-miR-129-5p did not show a significant association with either neuropathological staging parameter (Supplementary Table S3).

## Discussion

Our study represents the first independent assessment of 10 miRNAs showing prior evidence of differential expression in post-mortem brain samples in previous work from our group for Parkinson’s disease^10^ and Alzheimer’s disease.^11^ To this end, we collected and analysed one of the largest case–control collections of Parkinson’s disease and Alzheimer’s disease post-mortem brain samples (total *n* = 458) available in the field. To our knowledge, our study is the first to highlight a significant downregulation of the Alzheimer’s disease candidate miRNA hsa-miR-129-5p in Parkinson’s disease brains compared with controls. As such, it is also the first to describe the shared signature of hsa-miR-129-5p expression in Parkinson’s disease and Alzheimer’s disease. In addition, for hsa-miR-132-3p/-5p—where we drastically increased the sample size of analysed Parkinson’s disease brain samples—we can now, for the first time, reliably conclude that hsa-miR-132-3p/-5p is not only downregulated in Alzheimer’s disease but also in Parkinson’s disease brains (for details on the published literature of these miRNAs in Parkinson’s disease, see Supplementary Note). Furthermore, to the best of our knowledge, our study is the first to assess the association of hsa-miR-132-3p/-5p and hsa-miR-129-5p brain expression with respect to Parkinson’s disease neuropathology. These analyses showed that hsa-miR-132-3p/-5p (but not miR-129-5p) is significantly associated with α-synuclein Braak staging in Parkinson’s disease cases, suggesting that this miRNA may play a role in α-synuclein aggregation. These findings provide the basis for future *in vivo* or *in vitro* work on the role of hsa-miR-132-3p/-5p and hsa-miR-129-5p in Parkinson’s disease. Intriguingly, hsa-miR-132 may bind to *SNCA* mRNA as evidenced by publicly available next-generation sequencing-based high-throughput sequencing of RNA isolated by cross-linking immunoprecipitation data in the human brain.^24^ Further functional studies focusing on miR-132-3p/-5p and miR-129-5p need to elucidate the underlying Parkinson’s disease pathomechanisms while also considering possibly shared pathomechanisms with Alzheimer’s disease (also see Supplementary Note for previous knowledge on hsa-miR-132-3p/-5p and miR-129-5p).

The remaining seven miRNAs were either significantly dysregulated only in Alzheimer’s disease (hsa-miR-138-5p and hsa-miR-195-5p)^12^ or did not show differential expression in neither Parkinson’s disease nor Alzheimer’s disease brain tissue in our dataset (hsa-miR-133b, hsa-miR-497-5p, hsa-miR-628-5p, hsa-miR-125b-5p and hsa-miR-501-3p).^12^

There are several potential limitations in our study. First, while our data set represents one of the largest collection of post-mortem case–control brain samples in Parkinson’s disease, our sample size may still be underpowered to detect subtle differences in miRNA expression. As a result, some of the non-validations of the candidate miRNAs assessed in this study may represent false-negative findings. Second, similar to almost all other recent gene expression studies in the field, we used bulk tissue to quantify miRNA expression. Thus, the reported differential miRNA expression results may at least in part be the result of differences in cell-type composition of our sample rather than a *bona fide* downregulation of the miRNA on a cellular level. This limitation might become addressable by using single-cell/-nucleus sequencing, which was beyond the scope of this work. Third, compared with hsa-miR-132-3p, hsa-miR-132-5p has a rather low expression level in all tissues, based on expression data in the Human miRNA tissue atlas (https://ccb-web.cs.uni-saarland.de/tissueatlas). Whether this reflects a more limited pathophysiological role in Alzheimer’s disease and Parkinson’s disease remains to be investigated in further studies. Fourth, due to the origin of Parkinson’s disease and Alzheimer’s disease samples and corresponding controls that were recruited and contributed by two different centres in the UK, we could not directly compare the miRNA expression between the two diseases. For instance, miR-132-3p expression results (Table 1) suggest that the expression of this miRNA is lower in Alzheimer’s disease compared with Parkinson’s disease brains, but this may also be due to various other reasons, including differences in case ascertainment and sample handling between centres and/or different neuropathological stages. Therefore, it was not possible to probe for potential differences between the two diseases in the context of our study. For the same reason, it was not possible to pool the control samples of the two centres as they would have been compared against disease samples from only one centre. Lastly, as our study compared gene expression in post-mortem tissue of diseased individuals and unaffected controls, we cannot reliably distinguish cause–effect relationships regarding the role of hsa-miR-132-3p/-5p and hsa-miR-129-5p in Parkinson’s disease (or Alzheimer’s disease). Interestingly, the analyses using neuropathological α-synuclein Braak staging in Parkinson’s disease cases suggest that hsa-miR-132-3p/-5p may play a role in the progressive accumulation of α-synuclein along the disease course, beyond the initial disease stages. However, this does not exclude its involvement in pathomechanisms in early disease phases. In contrast, miRNA hsa-miR-129-5p did not show a significant association with neuropathological staging in Parkinson’s disease cases, possibly suggesting a role predominately at earlier disease stages.

Despite these limitations, we note that several lines of independent evidence support the role of both miR-132 and miR-129-5p in Parkinson’s disease and Alzheimer’s disease: For instance, in Parkinson’s disease, downregulation of the miR-132-3p/miR-212-3p cluster (the precursor hsa-mir-132 and precursor hsa-mir-212 share the same primary transcript)^25^ was reported to occur in α-synuclein (A30P)-transgenic mice (also see Supplementary Note).^26^ Secondly, it has been shown that deleting the miR-132/212 cluster in mouse models led to impaired memory^27^ and increased Aβ production as well as amyloid plaque formation.^28^ Third, both hsa-miR-132-3p and miR-129-5p emerged among miRNAs with neuroprotective roles against amyloid β-peptide accumulation in primary mouse and human neuronal cell culture models.^29^ Lastly, a neuroprotective role for miR-129-5p has recently been reported in a rat model of Alzheimer’s disease (established by injecting Aβ25-35 into the brain), where miR-129-5p inhibited neuronal apoptosis.^30^

## Conclusion

Our study provides novel and conclusive evidence that hsa-miR-132-3p/-5p and hsa-miR-129-5p are differentially expressed in post-mortem brain samples of both Parkinson’s disease and Alzheimer’s disease. Furthermore, for the first time, we provide evidence of an association between hsa-miR-132-3p/-5p expression in the brain and α-synuclein Braak staging in Parkinson’s disease. These data suggest that miR-132-3p/5p may play a role in disease progression. Future work needs to independently replicate our observations and to elucidate whether these co-occurring miRNA expression results are the result of shared pathogenic mechanisms across both these diseases.

## Supplementary Material

fcac274_Supplementary_Data

## Data Availability

All data generated or analysed during this study are available from the corresponding author on request.

## Abbreviations

GLM =: generalized linear model
HITS-CLIP =: high-throughput sequencing of RNA isolated by cross-linking immunoprecipitation
IQR =: interquartile range
miRNA =: microRNA
*N* =: number
PMI =: post-mortem interval
QC =: quality control
RIN =: RNA integrity number
SE =: standard error
STG =: superior temporal gyrus
