# Supplementary material for: Common signatures of differential microRNA expression in Parkinson’s and Alzheimer’s disease brains
Source: Brain Commun. 2022 Oct 28;4(6):fcac274. doi: 10.1093/braincomms/fcac274 (PMC9645562; doi:10.1093/braincomms/fcac274)
Supplement: fcac274_Supplementary_Data [file fcac274_supplementary_data.pdf]

Common signatures of differential microRNA expression in Parkinson’s and Alzheimer’s disease brains

Valerija Dobricic, Marcel Schilling, Ildiko Farkas, Djordje O Gveric, Olena Ohlei, Jessica Schulz, Lefkos Middleton, Steve M Gentleman, Laura Parkkinen, Lars Bertram, Christina M Lill*

***Corresponding Author:**

Dr. Christina Lill, MD, MSc, Translational Epidemiology Group, LIGA, Ratzeburger Allee 160, Haus V50, 1st floor, Room 203.1, 23562 Lübeck, Germany, telephone: +49 451 3101 7493, fax: +49 451 3101 7494, email: christina.lill@uni-luebeck.de

Supplementary Material

**Content**	**Page**
Supplementary Table 1	2
Supplementary Table 2	3
Supplementary Table 3	4
Supplementary Figure 1	4
Supplementary Note	5
Supplementary References	8

Supplementary Table 1. Overview of the Parkinson's disease case-control *post-mortem* brain samples analyzed in this study

	PD cases	PD controls
**Total number**	214	47
**Sex**		
Number of males (%)	144 (67.3%)	26 (55.3%)
p-value[a]	0.145	
**Age at death (years)**		
average (±SD)	78.8 (6.7)	81.1 (9.7)
median (IQR)	79 (75-84)	83 (75.5-89)
range	57-93	58-96
p-value[b]	0.137	
**Age at onset (years)[c]**		
average (±SD)	65.6 (10.2)	n.a.
median (IQR)	67 (59-72)	n.a.
range	30-88	n.a.
**Disease duration (years)[c]**		
average (±SD)	13.0 (7.3)	n.a.
median (IQR)	11 (8-17)	n.a.
range	1-44	n.a.
**PMI (hours)**		
average (±SD)	19.4 (9.5)	21.1 (9.7)
median (IQR)	19 (12-24)	21 (13.5-24.75)
range	2-48	5-48
p-value[b]	0.302	
**RIN value**		
average (±SD)	3.7 (1.4)	3.6 (1.4)
median (IQR)	3.05 (2.5-4.68)	2.8 (2.4-4.8)
range	2.2-7.5	2.3-7.6
p-value[b, d]	0.629	
**RNA A260/280**		
average (±SD)	1.94 (0.03)	1.95 (0.05)
median (IQR)	1.94 (1.92-1.95)	1.94 (1.93-1.96)
range	1.87-2.06	1.89-2.20
p-value[b]	0.130	
**Alpha-synuclein Braak stage[e]**		
Stage 3	15 (7.0%)	n.a.
Stage 4	21 (9.8%)	n.a.
Stage 5	46 (21.5%)	n.a.
Stage 6	120 (56.1%)	n.a.
Data unavailable	12 (5.6%)	n.a.
**Tau Braak stage[f]**		n.a.
Stage 0	9 (4.2%)	n.a.
Stage 1	35 (16.5%)	n.a.
Stage 2	146 (68.2%)	n.a.
Stage 3	20 (9.3%)	n.a.
Stage 5	2 (0.9%)	n.a.
Data unavailable	2 (0.9%)	n.a.
**Clinical dementia**		
Yes	73	0
No	63	1
Data unavailable	78	46

Legend. *Post-mortem* brain samples from the superior temporal gyrus of Parkinson's (PD) patients and corresponding controls were provided by the Parkinson's UK Brain Bank at Imperial College London. SD = standard deviation; IQR = interquartile range; PMI = *post-mortem* interval; RIN = RNA integrity number; [a] = Pearson's Chi-squared test with Yates'

continuity correction; [b] = Welch's two-sample t-test; [c] = data available for 155 individuals; [d] = due to some deviation from normality, we also performed Wilcoxon rank sum test, but results did not change substantially, thus only the results of Welch's two-sample t-test are provided here; [e] = data available for 202 individuals; [f] = data available for 212 individuals; n.a. = not applicable/not available.

Supplementary Table 2. Overview of the Alzheimer's disease case-control *post-mortem* brain samples analyzed in this study

	**AD cases**	**AD controls**
**Total number**	99	91
**Sex**		
Number of males (%)	50 (51%)	51 (56%)
p-value[a]	0.536	
**Age at death (years)**		
average (±SD)	81.6 (8.0)	77.5 (13.8)
median (IQR)	83 (77.5-87)	81(68.5-88.5)
range	61-95	41-100
p-value[b]	0.0142	
**PMI (hours)**		
average (±SD)	57.1 (30.6)	48.41 (31)
median (IQR)	48 (30-73.75)	48 (24-48)
range	9-140	5-168
p-value[b]	0.0540	
**RIN value**		
average (±SD)	3.0 (1.2)	4.2 (1.4)
median (IQR)	2.6 (2.3-3.25)	4.00 (2.95-5.2)
range	1.2-7.8	2.1-7.6
p-value[b, c]	6.92e-09	
**RNA A260/280**		
average (±SD)	1.90 (0.04)	1.93 (0.04)
median (IQR)	1.89 (1.86-1.94)	1.93 (1.91-1.95)
range	1.80 - 1.99	1.80-2.00
p-value[b, c]	2.063e-05	
**Braak Stage**		
Stage 0	0 (0%)	6 (6.6%)
Stage I/II	0 (0%)	72 (79.1%)
Stage III	0 (0%)	6 (6.6%)
Stage IV	8 (8.1%)	0 (0%)
Stage V/VI	91 (91.9%)	0 (0%)
Data unavailable	0 (0%)	7 (7.7%)

Legend. *Post-mortem* brain samples from the superior temporal gyrus of Alzheimer's (AD) patients and corresponding controls originate from the biobank of the longitudinal, prospective Oxford Project to Investigate Memory and Aging (OPTIMA) at University of Oxford (also described in Dobricic et al., 2021 [1]). SD = standard deviation; IQR = interquartile range; PMI = *post-mortem* interval; RIN = RNA integrity number; [a] = Pearson's Chi-squared test with Yates' continuity correction; [b] = Welch's two-sample t-test; [c] = due to some deviation from normality, we also performed Wilcoxon rank sum test, but results did not change substantially, thus only the results of Welch's two-sample t-test are provided here; n.a. = not available.

Supplementary Table 3. Linear regresion analysis of hsa-miR-132-3p/-5p and hsa-miR-129-5p on α-synuclein and tau Braak staging in *post-mortem* Parkinson's disease brain samples

MiRNA	Braak staging	Effect estimate (95% CI)	p
**hsa-miR-132-3p**	**α-synuclein**	**-0.211 (-0.352, -0.071)**	**3.51E-03***
	tau	-0.113 (-0.255, 0.028)	0.119
**hsa-miR-132-5p**	**α-synuclein**	**-0.185 (-0.327, -0.043)**	**0.0117***
	**tau**	**-0.166 (-0.309, -0.024)**	**0.0235**
hsa-miR-129-5p	α-synuclein	-0.077 (-0.230, 0.077)	0.329
	tau	-0.060 (-0.220, 0.100)	0.463

Legend. This table displays the linear regression results of hsa-miR-132-3p/-5p and hsa-miR-1129-3p/-5p on α-synuclein and tau Braak staging in *post-mortem* brain samples of 202 (α-synuclein) and 212 (tau) Parkinson's disease patients. P-values displayed in bold are at least nominally significant, * denotes p-values showing significant results after Bonferroni correction (α=0.0125), also see Methods.

Supplementary Figure 1. Box plot displaying the distribution of qPCR-based Ct values for Alzheimer's disease samples analyzed in this study

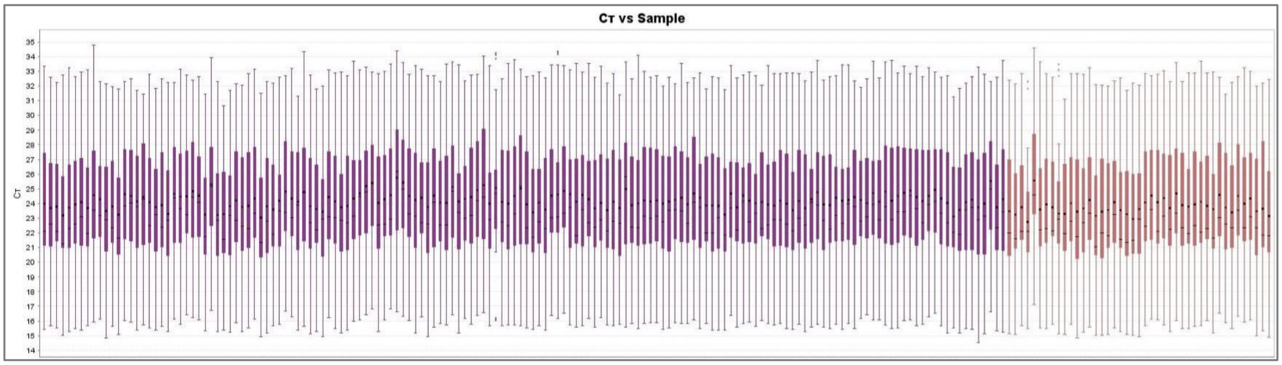

Legend. This figures shows similar distributions of Ct values in samples with lower (RIN <5, represented in purple color) vs higher RIN values (RIN ≥ 5, represented in brown color) values. The horizontal black bar shows the median Ct value. The black circle shows the mean Ct value. The solid box shows the range of the middle 50% of the Ct values for each sample. Note that this figures has already been published in Dobricic et al., 2021).[1]

Supplementary note. Summary of previous studies on hsa-miR-129-5p and hsa-miR-132-3p/5p in the context of Parkinson's disease following a systematic literature review

1. hsa-miR-129-5p

a) Differential expression of hsa-miR-129-5p in post-mortem Parkinson's disease and control brains: Sample sizes in previous studies were small with 11, 62, and 32 combined Parkinson's disease cases and controls, and none of the studies had reported hsa-miR-129-5p as differentially expressed.[2–4] Our previous meta-analysis combining these small studies was based on a total of 58 Parkinson's disease and 47 control brains (total n=105), and showed nominal significant differential downregulation in Parkinson's disease vs control brains (p=7.74E-04), which did not survive multiple testing correction.[5] After the data freeze of our previous meta-analysis, one more study including 8 Parkinson's disease and 8 control brains was published.[6] This study was included in the updated meta-analysis performed in the current study.

b) Association with Parkinson's disease neuropathology as marker of disease progression: We did not identify any studies reporting on the quantification of hsa-miR-129-5p levels in any tissue of Parkinson's disease patients (brain/blood/CSF) with Parkinson's disease neuropathology (Parkinson's disease Braak staging) in brain.

c) Quantifications of hsa-miR-129-5p in other human Parkinson's disease studies: Hsa-miR-129-5p was reported to be increased in peripheral blood lymphocytes in 18 treated vs 20 untreated Parkinson's disease patients.[7] A recent NGS-based miRNA screen in plasma samples of Parkinson's disease and control blood samples assessed association with clinical disease progression but did not report on individual miRNAs.[8]

d) Evidence from other in vitro and in vivo functional studies: We did not identify any *in vitro* nor *in vivo* studies assessing the role of hsa-miR-129-5p in Parkinson's disease cellular or animal models.

=> *Interpretation*: Our study is the first to report a significant downregulation of hsa-miR-129-5p in Parkinson's disease brain vs. controls and to assess the association of hsa-miR-129-5p expression in brain with neuropathological Parkinson's disease Braak staging. Functional studies investigating the molecular mechanisms of hsa-miR-129-5p are currently missing.

2. hsa-miR-132-3p/5p

a) Differential expression of hsa-miR-132-3p/5p in post-mortem Parkinson's disease and control brains: Sample sizes in previous studies on hsa-miR-132-3p and hsa-miR-132-5p were small with 10, 12, and 62 combined Parkinson's disease cases and controls [2,3,9] and with 9 and 62 combined cases and controls[2,3], respectively. Our previous meta-analysis for hsa-miR-132-3p combining these small studies was based on a total of 41 Parkinson's

disease and 43 control brains (total n=105), and showed significant differential downregulation in Parkinson's disease vs control brains (p=6.37E-05).[5] For hsa-miR-132-5p, data were insufficient to perform a meta-analysis.[2,3] After the data freeze of our previous meta-analysis, two more studies involving 25 (hsa-miR-132-3p)[10] and 16 (hsa-miR-132-3p and hsa-miR-132-5p)[6] combined case and control brains were published and included in the updated meta-analysis performed in the current study.

b) Association with Parkinson's disease neuropathology as marker of disease progression: We did not identify any study reporting association analyses of hsa-miR-132-3p/-5p expression in brain and Parkinson's disease neuropathology (Parkinson's disease Braak staging). Burgos et al. reported that hsa-miR-132-5p expression in CSF decreased with advanced Lewy body pathology in the brain, although this study was not limited to Parkinson's disease but also analyzed Alzheimer's disease cases and controls.[11] Furthermore, in serum samples of Parkinson's disease patients, expression of hsa-miR-132-3p (and hsa-miR-146-5p) was reported to be negatively correlated with Braak staging.[12]

c) Quantifications of hsa-miR-132-3p/5p in other human Parkinson's disease studies: As for hsa-miR-129-5p (see above), hsa-miR-132-3p was reported to be increased in peripheral blood lymphocytes of 18 treated vs 20 untreated Parkinson's disease patients.[7] A recent NGS-based miRNA screen in plasma samples of Parkinson's disease and control blood samples assessed association with clinical disease progression but did not report on individual miRNAs[8].

d) Evidence from other in vitro and in vivo functional studies: Hsa-miR-132-3p has been implicated in a variety of functional domains relevant for the human brain, including neuroprotection, memory, neural growth, synapse function, apoptosis, and inflammation (e.g., reviewed in ref. [13–15]). For Parkinson's disease, several different molecular mechanisms were suggested as a possible link between hsa-miR-132-3p/5p dysregulation and Parkinson's disease: In an cell model, hsa-mir-132-3p was reported to activate the SIRT1/p53 pathway resulting in neuronal death[16] and to downregulate GLRX with subsequent promotion of neuroinflammation[17]. Inhibition of hsa-miR-132-5p in both cell and mouse models reduced apoptosis and suppressed autophagy, presumably by targeting ULK1.[18] Finally, simultaneous downregulation of miR-212-3p and miR-132-3p was reported in alpha-synuclein (A30P)-transgenic mice.[19] It is important to emphasize that these reports still need to be validated independently.

=> *Interpretation*: Downregulation of hsa-miR-132-3p was reported in Parkinson's disease brains vs controls previously, but sample sizes were very small. Our study now confirms this finding using a much larger independent dataset. To the best of our knowledge, no previous study has investigated the association of hsa-miR-132-3p/5p gene expression in brain and neuropathological Parkinson's disease phenotypes, such as alpha-synuclein staging. Thus, our study is the first to report such an association for hsa-miR-132-3p/5p.

Functional evidence of hsa-miR-132-3p/5p points towards an important role of this miRNA in various neuronal processes; additional studies in Parkinson’s disease are needed to determine the exact underlying mechanisms.

Supplementary References

1. Dobricic V, Schilling M, Schulz J, et al. Differential microRNA expression analyses across two brain regions in Alzheimer's disease. *bioRxiv*. Published online May 31, 2021:2021.05.31.446406. doi:10.1101/2021.05.31.446406

2. Cardo LF, Coto E, Ribacoba R, et al. MiRNA Profile in the Substantia Nigra of Parkinson's Disease and Healthy Subjects. *J Mol Neurosci*. 2014;54(4):830-836. doi:10.1007/s12031-014-0428-y

3. Hoss AG, Labadorf A, Beach TG, Latourelle JC, Myers RH. microRNA profiles in Parkinson's disease prefrontal cortex. *Front Aging Neurosci*. 2016;8(MAR). doi:10.3389/fnagi.2016.00036

4. Tatura R, Kraus T, Giese A, et al. Parkinson's disease: SNCA-, PARK2-, and LRRK2- targeting microRNAs elevated in cingulate gyrus. *Park Relat Disord*. 2016;33:115-121. doi:10.1016/j.parkreldis.2016.09.028

5. Schulz J, Takousis P, Wohlers I, et al. Meta-analyses identify differentially expressed micrornas in Parkinson's disease. *Ann Neurol*. 2019;85(6). doi:10.1002/ana.25490

6. Schulze M, Sommer A, Plötz S, et al. Sporadic Parkinson's disease derived neuronal cells show disease-specific mRNA and small RNA signatures with abundant deregulation of piRNAs. *Acta Neuropathol Commun*. 2018;6(1):58. doi:10.1186/s40478-018-0561-x

7. Alieva AK, Filatova E V., Karabanov A V., et al. MiRNA expression is highly sensitive to a drug therapy in Parkinson's disease. *Park Relat Disord*. 2015;21(1):72-74. doi:10.1016/j.parkreldis.2014.10.018

8. Kern F, Fehlmann T, Violich I, et al. Deep sequencing of sncRNAs reveals hallmarks and regulatory modules of the transcriptome during Parkinson's disease progression. *Nat Aging*. 2021;1(3):309-322. doi:10.1038/s43587-021-00042-6

9. Sethi P, Lukiw WJ. Micro-RNA abundance and stability in human brain: specific alterations in Alzheimer's disease temporal lobe neocortex. *Neurosci Lett*. 2009;459(2):100-104. doi:10.1016/J.NEULET.2009.04.052

10. Xing RX, Li LG, Liu XW, Tian BX, Cheng Y. Down regulation of miR-218, miR-124, and miR-144 relates to Parkinson's disease via activating NF-κB signaling. *Kaohsiung J Med Sci*. 2020;36(10):786-792. doi:10.1002/kjm2.12241

11. Burgos K, Malenica I, Metpally R, et al. Profiles of extracellular miRNA in cerebrospinal fluid and serum from patients with Alzheimer's and Parkinson's diseases correlate with disease status and features of pathology. *PLoS One*. 2014;9(5). doi:10.1371/journal.pone.0094839

12. Shu Y, Qian J, Wang C. Aberrant expression of microRNA-132-3p and microRNA-146a-5p in Parkinson's disease patients. *Open Life Sci*. 2020;15(1):647-653. doi:10.1515/biol-2020-0060

13. Juźwik CA, S. Drake S, Zhang Y, et al. microRNA dysregulation in neurodegenerative diseases: A systematic review. *Prog Neurobiol*. 2019;182. doi:10.1016/j.pneurobio.2019.101664

14. Hernandez-Rapp J, Rainone S, Hébert SS. MicroRNAs underlying memory deficits in neurodegenerative disorders. *Prog Neuro-Psychopharmacology Biol Psychiatry*. 2017;73:79-86. doi:10.1016/j.pnpbp.2016.04.011

15. Qian Y, Song J, Ouyang Y, et al. Advances in roles of miR-132 in the nervous system. *Front Pharmacol*. 2017;8(OCT). doi:10.3389/fphar.2017.00770

16. Qazi TJ, Lu J, Duru L, Zhao J, Qing H. Upregulation of mir-132 induces dopaminergic neuronal death via activating SIRT1/P53 pathway. *Neurosci Lett*. 2021;740. doi:10.1016/j.neulet.2020.135465

17. Gong X, Huang M, Chen L. Mechanism of miR-132-3p Promoting Neuroinflammation and Dopaminergic Neurodegeneration in Parkinson's Disease. *eNeuro*. 2022;9(1). doi:10.1523/ENEURO.0393-21.2021

18. Zhao J, Yang M, Li Q, Pei X, Zhu X. MiR-132-5p regulates apoptosis and autophagy in MPTP model of Parkinson's disease by targeting ULK1. *Neuroreport*. 2020;31(13):959-965. doi:10.1097/WNR.0000000000001494

19. Gillardon F, Mack M, Rist W, et al. MicroRNA and proteome expression profiling in early-symptomatic α-synuclein(A30P)-transgenic mice. *Proteomics - Clin Appl*. 2008;2(5):697-705. doi:10.1002/prca.200780025
